# Local Acoustic Fields Powered Assembly of Microparticles and Applications

**DOI:** 10.3390/mi10120882

**Published:** 2019-12-16

**Authors:** Hui Shen, Kangdong Zhao, Zhiwen Wang, Xiaoyu Xu, Jiayu Lu, Wenjuan Liu, Xiaolong Lu

**Affiliations:** 1 State Key Laboratory of Mechanics and Control of Mechanical Structures, Nanjing University of Aeronautics and Astronautics, Nanjing 210016, Jiangsu, China; shenhui115@nuaa.edu.cn (H.S.); zhaokangdong@nuaa.edu.cn (K.Z.); wangzhiwen@nuaa.edu.cn (Z.W.); xxy@nuaa.edu.cn (X.X.); ljyljy@nuaa.edu.cn (J.L.); 2 College of Materials Science and Engineering, Nanjing Tech University, Nanjing 211816, Jiangsu, China

**Keywords:** micromachines, assembly, acoustic field, radiation, streaming, modulation

## Abstract

Controllable assembly in nano-/microscale holds considerable promise for bioengineering, intracellular manipulation, diagnostic sensing, and biomedical applications. However, up to now, micro-/nanoscopic assembly methods are severely limited by the fabrication materials, as well as energy sources to achieve the effective propulsion. In particular, reproductive manipulation and customized structure is quite essential for assemblies to accomplish a variety of on-demand tasks at small scales. Here, we present an attractive assembly strategy to collect microparticles, based on local acoustic forces nearby microstructures. The micro-manipulation chip is built based on an enhanced acoustic field, which could tightly trap microparticles to the boundaries of the microstructure by tuning the applied driving frequency and voltage. Numerical simulations and experimental demonstrations illustrate that the capturing and assembly of microparticles is closely related to the size of particles, owing to the vibration-induced locally enhanced acoustic field and resultant propulsion force. This acoustic assembly strategy can open extensive opportunities for lab-on-chip systems, microfactories, and micro-manipulators, among others.

## 1. Introduction

The controllable assembly of complex structures out of simple micro- and nanoparticles is of great importance for manufacturing micro and nanomachines [1,2,3,4]. In recent years, particles with different sizes, shapes, and compositions have been exploited to assemble a diverse set of configurations, including particle clusters, linear chains, and repeated arrays [5,6]. Those assemblies have attracted extensive attention owing to their considerable promise in catalysis, chemical or biosensing, cell biology, and tissue engineering [7,8,9,10,11]. To date, tremendous efforts have been devoted to study the assembly mechanisms, such as attractive or repulsive interactions and mechanical constraints, among others. Conventional and efficient organized assembly methods have been investigated for specific applications based on diverse physical or chemical effects, including the hydrophobic interactions and ionic interactions [12], templated dewetting [6], external electronic [13], magnetic field [14], and acoustic waves [15,16].

External fields have been developed as attractive strategies for accurate assembly of the particle components over a large area, demonstrating a combination of high productivity and facile operability. The external electronic or magnetic fields are based on dipole–dipole interactions, combining attractive or repulsive force together to create either one-dimensional chain structures or two-dimensional arrays [17]. However, these mechanisms have specific limitations on the particle materials, and thus are unable to work on non-magnetized or non-electrified materials. Acoustic waves are capable of precisely manipulating micro-objects regardless of the material properties [18,19,20,21,22]. In particular, Collins et al. demonstrated that interference between micro channel interfaces and a surface acoustic wave (SAW) could be used for generating fringe patterns of microparticles [23]. Dual et al. demonstrated numerous yeast cell trapping and hollow particles repulsion from oscillations of sharped edges in the acoustic field [24]. Other recent advances in acoustofluidics have made it possible to manipulate or trap microparticles with topographical structures [25,26,27]; however, the assembled structures cannot be precisely controlled. Hence, it is still highly desired to develop novel methods to achieve the easy, controllable, nonspecific, and reproducible performance of the organized assembly.

Here, we present an attractive manipulation methodology for assembling dispersed microparticles based on local acoustic fields near V-shape microstructures. This new assembly method relies on the localized acoustic propulsion forces generated around arrays of V-shape micropillars enclosed in a micro-manipulation chip, which are capable of propelling nearby microparticles and trapping them to attach to the narrow opening ends of micropillars and then assemble to a specific structure. Numerical simulations are used to study the local acoustic field in the presence of ultrasound vibration. The analytical results reveal that the assembly process is dominated by the first-order acoustic radiation force and second-order acoustic streaming force. Such an assembly method with high efficiency and controllability to microparticles offers new alternatives for manufacturing micromachines for biological engineering, lab-on-chip architectures, as well as nanoelectromechanical systems, among others.

## 2. Materials and Methods

### 2.1. Working Principles

**Figure 1:**
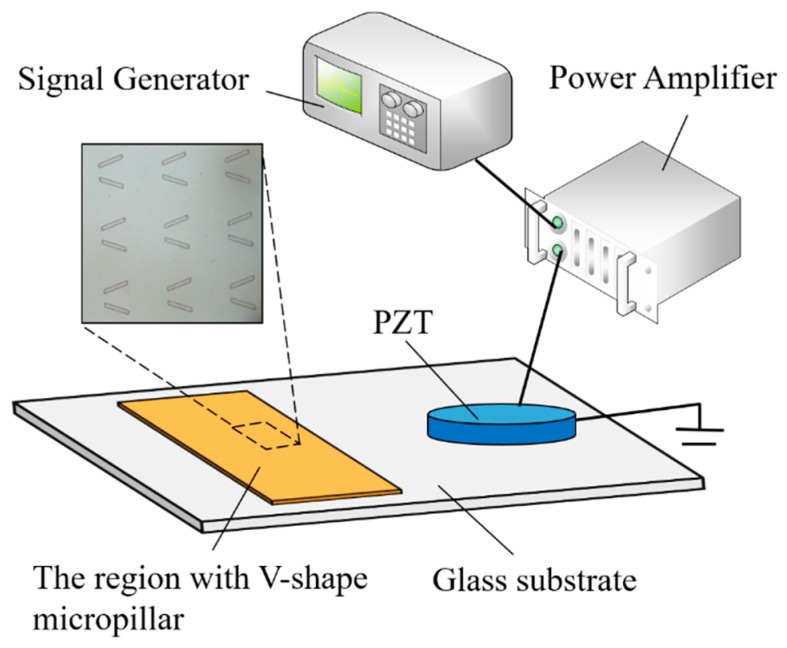
Schematic illustration of a micro-manipulation chip powered by the acoustic field.

The acoustic micro-manipulation chip comprises one piezoelectric transducer and one glass substrate, as illustrated in Figure 1. One piezoelectric (PZT) plate (PZT-8, Haiying Enterprise Group, Wuxi, China) with 0.5 mm thickness is utilized as the transducer and bonded with the 0.5 mm rectangular substrate (25 × 15 mm^2^) by the epoxy resin. Driven by a power amplifier coupled with a signal generator, the piezoelectric transducer could excite the vibration of glass substrate, thereby activating the organized assembly for microparticles in the fluidic chamber. The fluidic chamber made of polydimethylsiloxane (PDMS) elastomer is fabricated with an inner rectangular space (2000 × 1000 × 200 μm^3^) and bonded to the substrate over 80 °C heating for 30 min. Arrays of V-shape-like micropillars (see Figure 1 inset) fabricated on top of the micro-manipulation chip are made of photoresist SU-8 by ultraviolet (UV) photolithography and covered with one 100 nm layer of SiO_2_ by physical deposition. Consequently, using this facile methodology, the configuration of micropillars could be tailored via modifying photoetching parameters. Each side of the V-shape-like pillar is 1.4 × 10 × 60 μm^3^ (height × width × length) and the V-shape angle is 40°, with 30 μm and 75 μm width for the narrow and wide opening ends, respectively, which were designed to improve the stability during the fabrication and the capturing capability for microparticles. Compared with triangle style microstructures with single sharped trapping ends [24], the V-shape-like pillar could provide two neighbored trapping points at the narrow opening end for assembling two microchains simultaneously. The array spacing for micropillars is around 100 μm owing to the compromise between the trapping efficiency and interference of different microstructures. Being different from big fringe patterns generated around micro channels [23], microparticles trapped to the nearest pillar could be assembled to individual chains, allowing for precise control and local manipulation.

The working principle was proposed based on the system design shown in Figure 1. Microparticles were introduced to the manipulation chamber and then covered by a glass slide. These particles sedimented to the bottom of the manipulation cell and uniformly distributed over the substrate. This allows considerable amounts of microparticles to be widely dispersed over a large area and then simultaneously captured using active trap arrays equally distributed over the capture region. The ultrasound vibration of the glass substrate generates a localized microstreaming around the micropillar. Thus, all microparticles passing close to the pillars are driven by the resultant acoustic propulsion force, which is closely related to the particle size. By tuning the ultrasound parameters, trapped microparticles could be assembled by the radiation force to organized structures, for example, a flexible chain.

**Figure 2:**
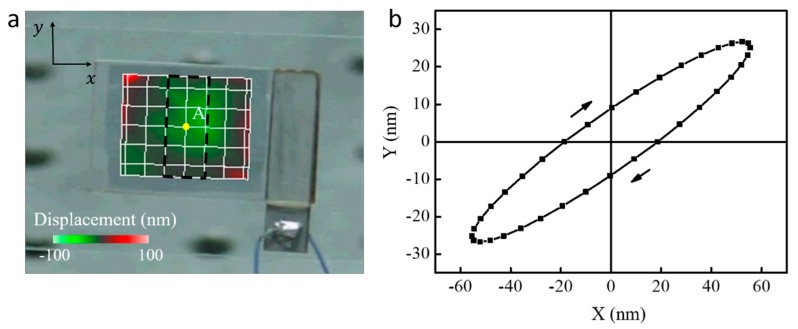
Vibration characterization of the acoustic micro-manipulation chip. (**a**) Vibration patterns of the acoustic micro-manipulation chip, and (**b**) moving trajectories of the measured point A.

The vibration characteristic of the acoustic manipulation chip was measured by a three-dimensional laser Doppler scanning vibrometer system (PSV-500-3D, Polytec GmbH, Waldbronn, Germany) and the results shown in Figure 2. Figure 2a illustrates the resonant vibration mode of the V-shape manipulation region occurring at 219.4 kHz, and the vibration magnitude of the glass substrate is 100 nm. The vibration mode is basically determined by the geometrical size of the substrate rather than the presence of the micropillars. One fixed point (point A) located at the center of micropillar area covered on the glass substrate is selected to analyze the vibration trajectory, which is plotted in Figure 2b. It can be observed that point A possesses a clockwise (cw) elliptical motion, which could be expressed as

(1) $$\{\begin{array}{c} v_{a}=\omega d_{a}e^{i\omega t}\\ v_{b}=-i\omega d_{b}e^{i\omega t} \end{array},$$

where ω is the angular frequency and $d_{a}$ (~60 nm) and $d_{b}$ (~30 nm) are the vibration amplitudes in *x* and *y* directions, respectively.

### 2.2. Theoretical Analysis

In accordance with the perturbation theory [28], liquid state equations under the external acoustic actuation can be described by the following variables: temperature *T*, pressure *p*, and velocity *v*. These variables can be expressed using the initial value, and first- and second-order (subscripts 0, 1, and 2, respectively) variables as

(2) $$\{\begin{array}{l} T=\;T_{0}+T_{1}+T_{2}\\ P=\;P_{0}+P_{1}+P_{2}\\ v=\;0+v_{1}+v_{2} \end{array},$$

where $T_{0}$ and $P_{0}$ are the constant temperature and pressure, respectively, before the presence of acoustic waves.

For these experiments in water, the thermal effect in the first-order equations is very small, thus it can be neglected to simplify the next analysis. The parameter averaged over a full vibration period (denoted as < >) satisfies the second-order continuity equation and Navier–Stokes equation.

(3) $$\{\begin{array}{c} \rho_{0}\nabla \cdot \langle v_{2}\rangle =\;-\nabla \cdot \langle \rho_{1}v_{1}\rangle \\ \eta \nabla^{2}\langle v_{2}\rangle +\beta \eta \nabla \left(\nabla \cdot \langle v_{2}\rangle \right)-\langle \nabla p_{2}\rangle =\langle \rho_{1}\partial_{t}v_{1}\rangle +\rho_{0}\langle (v_{1}\cdot \nabla )v_{1}\rangle \end{array},$$

where *η* is the dynamic viscosity and *β* is the viscosity ratio.

### 2.3. Numerical Simulations

**Figure 3:**
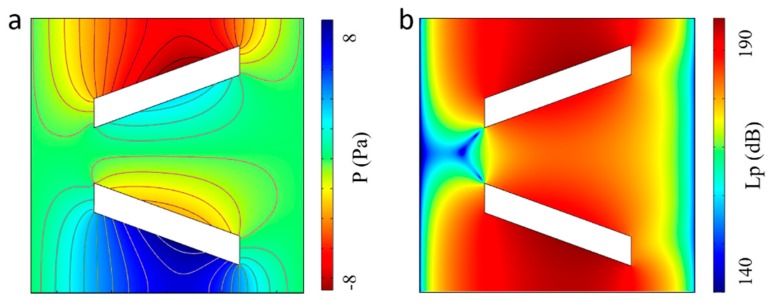
First-order acoustic pressure distribution induced by the elliptical motions of the micropillar. (**a**) The first-order sound pressure, and (**b**) the first-order sound pressure level.

**Figure 4:**
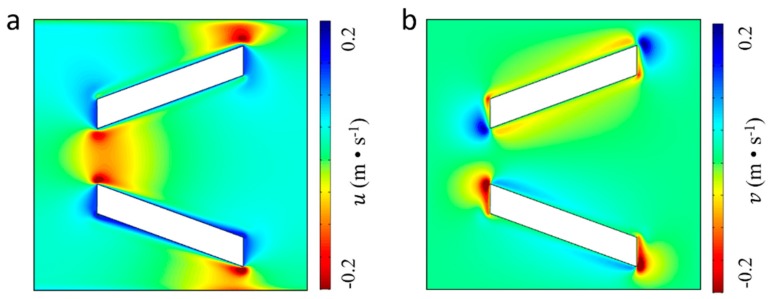
First-order acoustic velocity distribution induced by the elliptical motions of the micropillar. (**a**) The first-order velocity component in the *x* direction, and (**b**) the first-order velocity component in the *y* direction.

The finite element analysis software COMSOL (COMSOL AB, Stockholm, Sweden) with superior performance in simulating coupled multi-physical fields is used to solve acoustic streaming fields expressed by Equation (2). To obtain the vibration induced local acoustic streaming around the micropillar, the simulation domain is restricted to a small plane (100 × 100 μm^2^) without any external flow. In addition, measured moving trajectories of the micropillar are defined as the vibration boundary conditions according to Equation (1). Figure 3a illustrates the pressure distribution of first order acoustic field around a micropillar generated by the clockwise elliptical motion. According to the contour lines, the first order acoustic pressure is nearly symmetrical to both sides of V-shape pillars and decreases to a minimal value (~0 Pa) at the ends of each side. The remarkable change of the pressure gradient along each side of pillar indicates that particles could be trapped by strong acoustic radiation force at the end of the micropillars. Besides, it can also be obtained from Figure 3b that the acoustic pressure level in the *xy* plane is comparatively small in the narrow opening of the V-shape micropillar, indicating that the assembly of particles would be accomplished at the narrow opening part of the micropillars. Furthermore, the simulation results about the vibration induced first-order acoustic velocity field in in *x* and *y* directions are shown in Figure 4a,b, respectively. It can be noticed that the first-order acoustic velocity component in the *x* direction distributes symmetrically and the velocity component in the *y* direction distributes anti-symmetrically, which is caused by symmetrical geometry feature of pillar’s two sides along the *x* direction. In addition, there is a drastic variance at the end part of the micropillar and the highest value of the first-order acoustic velocity is 0.2 m/s.

**Figure 5:**
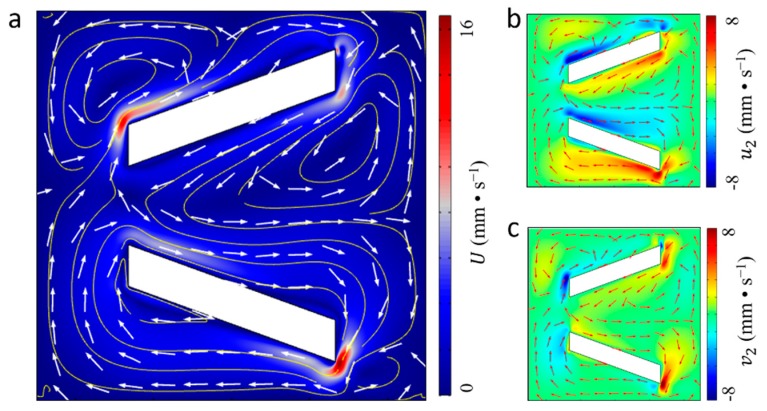
Second-order acoustic streaming fields excited by the clockwise elliptical motions of the micropillar. (**a**) The second-order streaming velocity in the *xy* plane, (**b**) the second-order streaming velocity in the *x* direction, and (**c**) the second-order streaming velocity in the *y* direction.

The calculated first-order oscillation velocity is then converted to the mass source term and body force term based on Equation (3) for solving the second-order acoustic streaming field using the weak-form finite element implementation. The calculated second-order acoustic streaming in Figure 5a displays dual clockwise rolls around sides of the V-shape micropillar, accompanied by several small counter clockwise rolls, corresponding to the cw elliptical motion of the micropillar shown in Figure 2b. The magnitude of the cw rolls is comparatively high near the end region of the pillar, with a maximum value of 16 mm/s. Moreover, the second-order acoustic streaming in the *x* direction and *y* direction (see Figure 5b,c, respectively) illustrates that the intensity of local acoustic streaming has a symmetrical distribution on both sides of the pillar. Also, the enhancement of closed-loop acoustic streaming around the sides of micropillar could provide sufficient propulsion force for driving microparticles to move towards the end parts of the micropillar for organized assembly. To achieve efficient capture performance, the concentration of microparticles should be relatively high to assure the probability for assembling microparticles around parallel micropillars. Above all, the numerical simulation results basically explain the mechanism of how to utilize the micropillar’s vibration-induced local enhanced acoustic field for the organized assembly of microparticles.

## 3. Experimental Results

### 3.1. Experimental Setup

**Figure 6:**
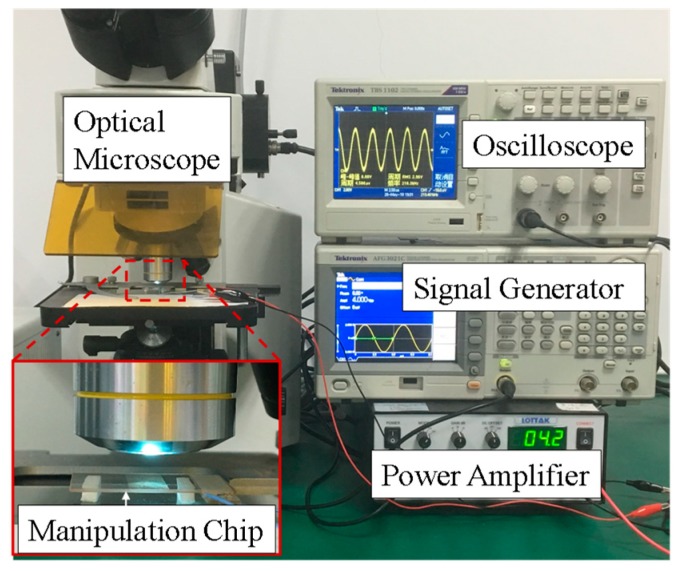
Experimental setup.

To demonstrate the aforementioned working mechanism, an experimental setup was built as displayed in Figure 6. The acoustic manipulation chip is placed on the stage of an optical microscope and the experiments are performed in the cell containing V-shape micropillars on the manipulation chip. The glass substrate vibrates at resonance, which could be adjusted by tailoring the input frequency and voltage, and then a localized microstreaming around the micropillars could be formed to manipulate microparticles around them. The oscilloscope is used to analyze the applied driving signals, and the ultrasound parameters including the frequency and voltage are tuned from the signal generator. The assembly process is monitored in a bright field method, using an upright optical microscope coupled with a CCD (Charge-coupled Device) camera (35 frames per second). For each set of experiments, the operation performance is recorded for 30 s.

### 3.2. Chain-Like Assembling of Micro Particles

**Figure 7:**
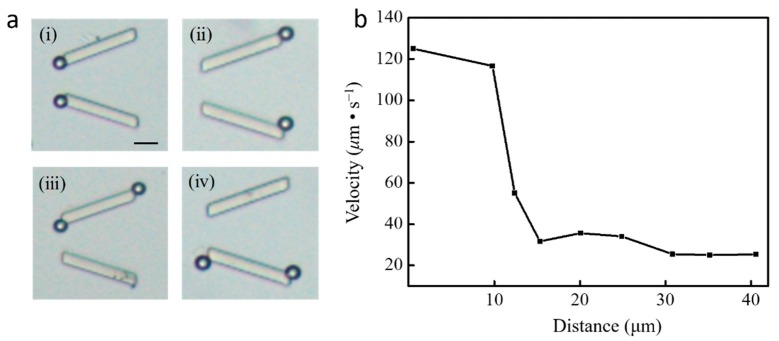
Actual microscopy images of single particle captured by the end of the V-shape micropillar. (**a**) Single particle captured by micropillar, and (**b**) velocity of particle at different distances.

**Figure 8:**
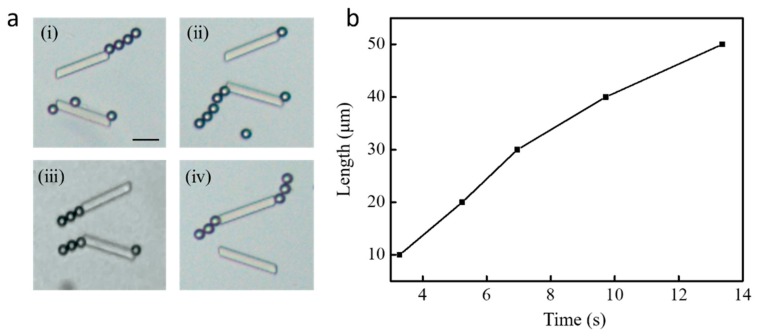
Chains assembly of particles trapped by the end of the V-shape micropillar. (**a**) Actual microscopy images of assembled chains (i and ii, chain in one side; iii, chains in same opening; iv, chains in same side), and (**b**) dependence of the length of particle chain upon the operating time.

Driving by the ultrasound power system, the micro-manipulation chip was tested for assembling polystyrene (PS) particles, which exhibit a high motion uniformity in a wide range of different size scales in aqueous solution. First, a 3 µl droplet of aqueous solution containing magnetic polystyrene microspheres was introduced for the following experiments. Figure 7a shows the actual microscopy images, in which several particle individuals (10 μm) are captured at each side end of the micropillar, at the acoustic frequency of 219.4 kHz and constant voltage of 15 V. The PS particles were instantaneously attracted toward the closest micropillar trap position and captured tightly to the pillar end. It can also be noticed that microparticles are normally trapped in the left narrow opening, which agrees well with the pressure field distribution in Figure 3b. Additionally, the experimental results in Figure 7a-ii show some particles captured at the right end, which may be caused by the scattering effect modified acoustic field with the presence of microparticles trapped around the micropillar [29]. Figure 7b shows the dependence of the microparticles’ velocity upon the distance to the micropillar. It is observed that the velocity is not constant and the peak velocity is around 125 μm/s. The microparticle out of the microstreaming area (distance ≥ 15 μm) is slowly accelerated to its steady speed (~25 μm/s) owing to the weak drag force from the far field of the ambient streaming introduced by the bulk acoustic wave vibration shown in Figure 2a. Upon reaching the vicinity of the micropillar (distance < 15 μm), the microparticle is rapidly accelerated because of the local enhanced acoustic streaming and then trapped by the acoustic radiation force at the pillar end. After 15 s operation with the same ultrasound parameters, an increasing number of PS particles will be trapped and assembled to the pillar end. The profiles of Figure 8a indicate that the multiple 10 μm microparticles can be assembled to a long chain from the initial dispersed elements after trapping. It is also observed that the chain of microparticles attaches to the pillar tightly with different angles and varies the length from three to five particles. The strong acoustic streaming force and radiation force acting on the particles are sufficient to maintain their organized assembling structures. Multiple microparticle elements assembled to form a straight chain around a pillar hold accurately linear position, as demonstrated in Figure 8b. Obviously, the length of chain is proportional to the time and the length of five-particle formed chain in 13.5 s is approximately 50 μm.

The proposed method now is valid for the region (3 × 10 mm^2^) marked out by the black dashed line shown in Figure 2a, which possesses elliptical moving trajectories. Thousands of V-shape micropillars with spacings of 100 μm fabricated in this region exhibit similar assembling behaviors, as shown in Figure 8a. It cannot be scaled up to other regions vibrating at different trajectories on the manipulation chip. According to the research from Henrik Bruus [30], the cross-over particle diameter at 219.4 kHz for the dominance between the acoustic streaming force and radiation force is around 6 μm. If the particles are smaller than 6 μm, the assembling strategy mentioned here would become less effective owing to the weakened radiation force. Under this condition, the acoustic streaming around oscillated microstructures would dominate micro or nano particles’ movement, which have been proven to realize the controllable manipulation, such as rotating cells and organisms [31], microbead-based enhancement of molecular exchange [32], and bi-directional transportation of micro-agents [33], among others.

**Figure 9:**
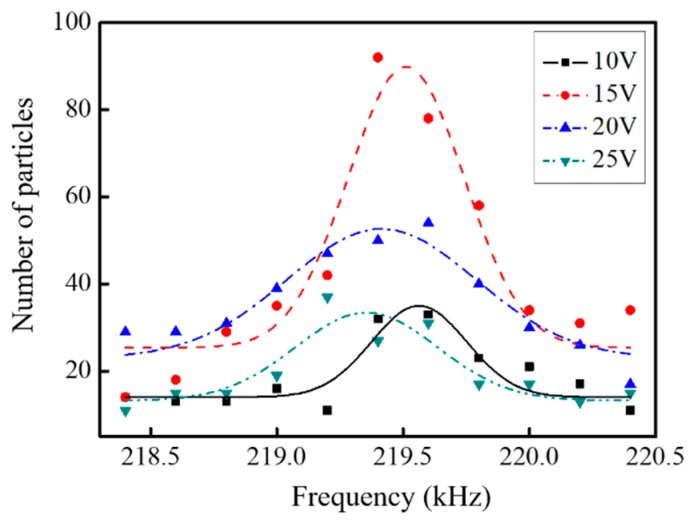
Influence of the ultrasound frequency on the assembling number of 5 μm polystyrene (PS) particles at different voltages.

**Figure 10:**
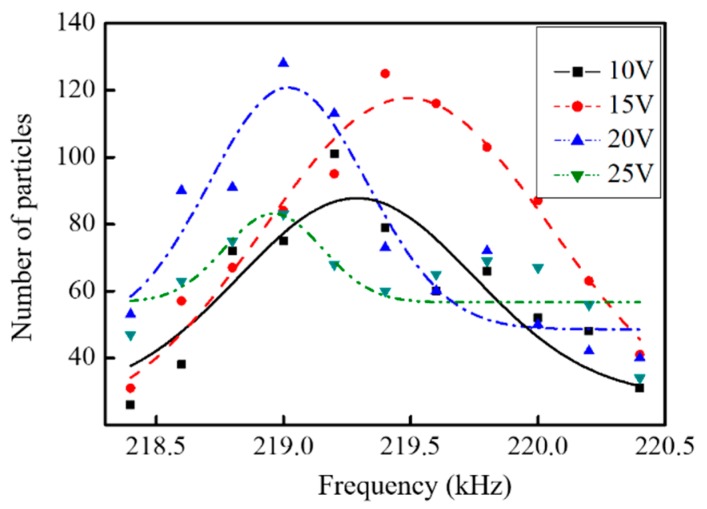
Influence of the ultrasound frequency on the assembling number of 10 μm PS particles at different voltages.

In consideration of the size effect on organized assembly performance, magnetic PS particles with different diameters (5 and 10 μm) are selected to measure the number of assembled particles under different driving conditions. The assembling number is defined as the number of microparticles assembled to chains in the entire microscopic field. Figure 9 and Figure 10 display the dependence of the assembling number of different particle sizes (5 and 10 μm) upon the driving frequency (between 218.4 and 220.4 kHz) at different driving voltages (between 10 and 25 V), respectively. Figure 9 illustrates that there is a noticeable variance in the assembling number over the examined frequency range. To be specified, at 219.4 kHz, all particles display a high assembling number, regardless of the different driving voltages, indicating that the manipulation chip works at resonant mode and the intensity of the acoustic field is highest in this frequency range. It can also be noticed that the assembling number increases dramatically from 10 to 15 V and decreases after 15 V, along with a maximum of 90 at 219.5 kHz and 15V driving voltage. As both acoustic radiation force and acoustic streaming force scale proportionally with the applied power [34,35], the geometrical effects of the assembled chains on the acoustic fields cannot be ignored. With the increasing length of chains, the distance between particles at the chains’ end and micropillar would increase, and thus the radiation force applied on those particles would decrease. Therefore, the drag force by the acoustic streaming would overcome the radiation force applied on particles at the chains end, leading to a limited length for the assembled chains, even at a high driving voltage.

In contrast, larger particles (10 μm) are less sensitive to frequency and voltage, as demonstrated in Figure 10. All manipulation results at different driving voltages exhibit a high assembling number over a relatively large frequency range, and there is a slight shifting for the optimal frequency at different voltages. Additionally, at these conditions, the assembling number of 10 µm particles is increased to 120, confirming that the capability for organized assembly can be increased by increasing the size of particles. There is a slight difference of preferential capture frequency for different particles during the experiments, which may be caused by the volume and shape variance of the droplet during the implementation. It is known that the radiation force is proportional to the particle’s radius cubed ($F\propto r^{3}$) [34], indicating that more big particles will be captured with a higher driving voltage at resonance. With the magnetic coating layers covered outside, the microparticles aggregated at the end of the V-shape micropillars would be self-adjusted to form a chain-like structure.

### 3.3. Control of Chain-Like Flexible Robotic Fingers

**Figure 11:**
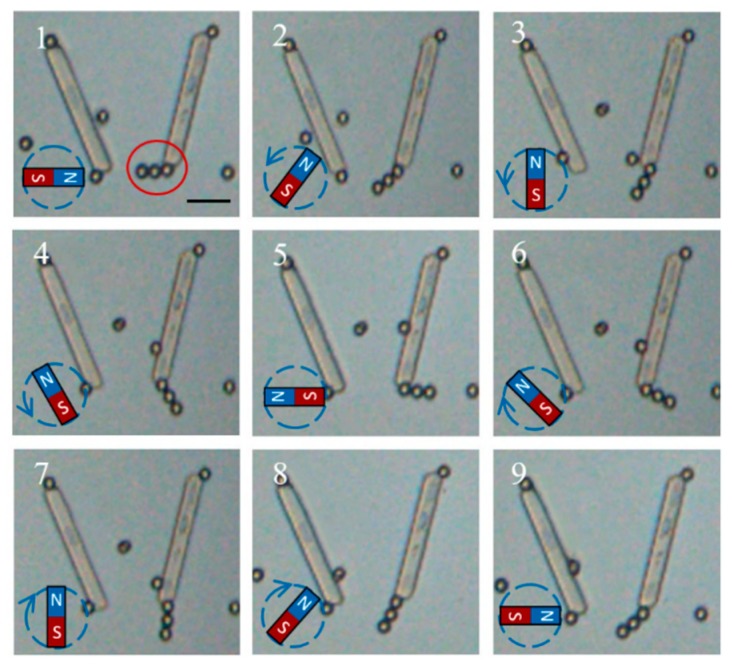
Image sequences showing the magnetic field enabled swing motion of a flexible finger acoustically assembled from 5 μm magnetic particles (1, A chain-like finger achieved by applying the acoustic field; 2 to 5, the flexible finger gradually swings to the right side by counter-clockwise rotating the magnetic field; 6 to 9, the swing motion reversed by clockwise rotating the magnetic field).

The proposed organized assembly method based on the acoustic fields around micropillars could be used for assembling particle elements made of materials with a higher acoustic impedance than water to acoustic field minima. Those particles with lower acoustic impedance than water would be driven to acoustic field maxima, which is not taken into consideration here [24]. In comparison with prior acoustic patterning studies, this method could capture particle elements to different local micropillar regions to form separate and reproducible assemblies, which is beneficial for precise manipulation in micrometer scale. In addition, microspheres bounded with different chemical functional groups could be acoustically assembled to complex functional features and controlled by other external fields. The image sequences demonstrated in Figure 11 display the chain-like flexible robotic fingers assembled from magnetic PS microspheres (5 μm) in the presence of both ultrasound and magnetic fields. The simultaneous application of the magnetic fields (while the ultrasound power is on) provides a net magnetization that enables on-demand alignment and reversible guidance to the flexible fingers. The alternating magnetic field gives rise to the motion of the finger to be periodically reoriented. Originally, the magnetic polystyrene microspheres are assembled to a chain-like finger pointing to the left direction, solely achieved by applying the acoustic field (Figure 11-1). Then, the flexible finger gradually swings to the right side, after counter-clockwise rotating the magnetic field (Figure 11-2–5). Moreover, the swing motion can be reversed by clockwise rotating the applied magnetic field (Figure 11-6–9). Compared with the controlled movement of the assembled flexible fingers, the aggregation of magnetic PS microspheres has no significant response towards the magnetic field. The switchable behavior of the flexible finger observed in Figure 11 demonstrates the crucial role of the magnetic microparticles in controlling the motion of organized functional structures with the external field. These results clearly indicate that functional microsystems acoustically assembled from magnetic elements could dynamically reorient or transform into different structures by tuning the external magnetic field.

## 4. Conclusions

In general, we developed an attractive strategy to utilize local acoustic fields near the microstructures for organized assembly of microspheres to obtain functional microsystems, for example, flexible chains that can serve as robotic arms or fingers. The acoustic fields around the V-shape micropillars are analyzed via numerical simulations. The analytical results reveal that the local acoustic field is determined by the geometry of the microstructures, in which the first-order minimum acoustic pressure occurs at pillar’s opening end part, while the second-order acoustic streaming is reinforced at the pillar’s sides. Hence, microparticles would be driving by the acoustic streaming propulsion to move along the micropillar’s side towards the pillar narrow opening ends and then trapped by the intensified acoustic radiation force. Multiple trapped microparticles will be aligned to a straight chain assembly spontaneously, without the necessity of extra feedback operations. The experimental results basically confirm the proposed working mechanism by using an array of V-shape micropillars to perform organized assemblies. Finally, the compatibility of the acoustic assembling method with other types of external fields is also discussed to explore potential applications. Combined with the modulation of external magnetic fields, one flexible chain acoustically assembled from magnetic microparticles could serve as a robotic finger with tunable orientations. The promising capabilities of organized assembly of microelements based on acoustic fields can open new possibilities for diverse applications such as autonomous micromachine systems, lab-on-chip systems, and microfacotries, among others.
